# The CroMyop study: myopia progression in Croatian children and adolescents—a 15-year retrospective analysis

**DOI:** 10.3389/fmed.2024.1405743

**Published:** 2024-05-31

**Authors:** Ana Maria Varošanec, Leon Marković, Zdenko Sonicki

**Affiliations:** ^1^University Eye Department, University Hospital “Sveti Duh”, Zagreb, Croatia; ^2^Reference Center of the Ministry of Health of the Republic of Croatia for Pediatric Ophthalmology and Strabismus, Zagreb, Croatia; ^3^Reference Center of the Ministry of Health of the Republic of Croatia for Inherited Retinal Dystrophies, Zagreb, Croatia; ^4^Faculty of Dental Medicine and Health Osijek, University Josip Juraj Strossmayer in Osijek, Osijek, Croatia; ^5^Department of Medical Statistics, Epidemiology and Medical Informatics, Andrija Štampar School of Public Health, University of Zagreb, School of Medicine, Zagreb, Croatia

**Keywords:** children and adolescents, cycloplegic refraction, refractive error, public health, myopic progression, myopia

## Abstract

**Purpose:**

Myopia is a major global health issue, especially among children and adolescents. Understanding its traits and progression is vital for proper management and prevention. This study aimed to fill a gap in research by analyzing demographic and refractive data concerning myopia among children and adolescents in Croatia, with the goal of providing insights into myopia prevalence, progression rates, and associated risk factors within the Croatian population.

**Design:**

This retrospective study utilized a comprehensive dataset from pediatric ophthalmology clinics at the University Eye Department, University Hospital “Sveti Duh,” Zagreb, Croatia. The dataset included electronic medical records spanning from January 2008 to July 2023, encompassing demographic and refractive data.

**Methods:**

Data analysis focused on individuals aged 4 to 18 years who were diagnosed with primary myopia and/or compound myopic astigmatism. Ophthalmic examinations, including visual acuity tests, cycloplegic refraction, and assessments for eye comorbidities, were conducted by experienced pediatric ophthalmologists. Statistical analysis, including t-tests, survival analysis, and logistic regression, was performed to assess myopia prevalence, progression rates, and associated factors. These analyses were adjusted for covariates such as age, parental myopia, and gender.

**Results:**

The study included 895 individuals, 51 premyopes, 813 low myopes, and 31 high myopes. The average age of diagnosis was 11.37 ± 3.59 years for premyopes, 11.18 ± 3.53 years for low myopes, and 11.44 ± 4.35 years for high myopes. The fastest progression occurred in 2021 and 2022, −0.5 ± 0.12 D/y for premyopes and − 0.45 ± 0.1 D/y for low myopes. Premyopic progression to low myopia was associated with age 7–9 years (HR 2.42, 1.53 to 3.21) and both parents being myopic (HR 920.27. 850.16 to 950.53). Low myopic individuals with both myopic parents displayed the fastest 11–24 months after first visit progression rates, −0.69 (−0.52 to −0.87) D/y, while the 7–9 age group demonstrated −0.36 (−0.24 to −0.45) D/y. Low myopes aged 7–9 years with baseline SE between −6 D and −4 D were more strongly associated with ≤ − 0.5 D progression (OR = 2.0, 95% CI −1.00 to 2.39).

**Conclusion:**

This study highlights the importance of environmental factors, genetics, and age in addressing myopia progression among Croatian youth, urging further research for effective local intervention strategies.

## 1 Introduction

Myopia has become one of the fastest-growing eye health challenges of the 21st century (1). Over the past 50 years, the worldwide prevalence of myopia has increased dramatically (2). Myopia stands as the predominant cause of correctable visual impairment across both adults and children in developed nations. Moreover, it emerges as a primary contributor to avoidable blindness in developing regions, underscoring its significant impact on global eye health (3). According to the World Health Organization’s findings in 2019, over 2.2 billion individuals are affected by vision impairments, with a staggering 1 billion suffering from preventable visual challenges (4). Increasing degrees of myopia put people at greater risk of sight-threatening complications, such as myopic maculopathy, retinal detachment, glaucoma, and cataracts (5). High myopia can cause pathological changes in the retina, choroid, and vitreous, leading to serious complications such as retinal detachment and neovascularization, macular hemorrhaging, and macular degeneration (6). The higher the degree of myopia, the greater the possibility of complications (blindness can result in severe cases) (5). Retinoscopy under cycloplegia is widely regarded as the gold standard for refractive assessment in children, owing to its ability to minimize the influence of accommodation, thus providing more accurate results (7). Considering the significant burden of the disease and its complications, tackling myopia becomes imperative (1). Timely identification of myopia progression patterns is the leading requisite for myopia practice as it aids in the decision of appropriate management (8). International Myopia Institute (IMI) introduced the concept of premyopia, which is defined as a refractive state of an eye of ≤ + 0.75 D and > −0.50 D in children where a combination of baseline refraction, age, and other quantifiable risk factors provide a sufficient likelihood of the future development of myopia to merit preventative interventions (9). Myopia progression has been analyzed in various studies (10–13), but only a few studies have been conducted in Europe (14).

The aims of this study were to analyze demographic and refractive characteristics, as well as progression patterns, of premyopia, low myopia, and high myopia among children and adolescents diagnosed and followed up in Croatia.

## 2 Materials and methods

### 2.1 Study population

This retrospective study retrieved medical electronic data files of children and adolescents diagnosed with refractive error and followed up in the pediatric ophthalmology clinics of the University Eye Department of the University Hospital “Sveti Duh” within the period from 1 January 2008 to 1 July 2023 in Zagreb, Croatia. The data contained the date of birth, date of first and follow-up visits, history of parental myopia, gender, sphere and cylinders of cycloplegic refraction and spectacle correction for both eyes, corrected and uncorrected visual acuity at near and at a distance for a single and both eyes. Ophthalmic examinations were conducted by experienced pediatric ophthalmologists. Children and adolescents from Central and Southeastern Europe, aged ≥4 and < 19 years, diagnosed with primary myopia and/or compound myopic astigmatism with at least 2 visits separated by at least 6 months were included. Inclusion criteria were children aged ≥4 years and < 19 years at the date of myopia diagnosis. Children aged <4 years were excluded based on their small number and since myopia etiology is considered different for newborns and toddlers (15).

**Figure 1:**
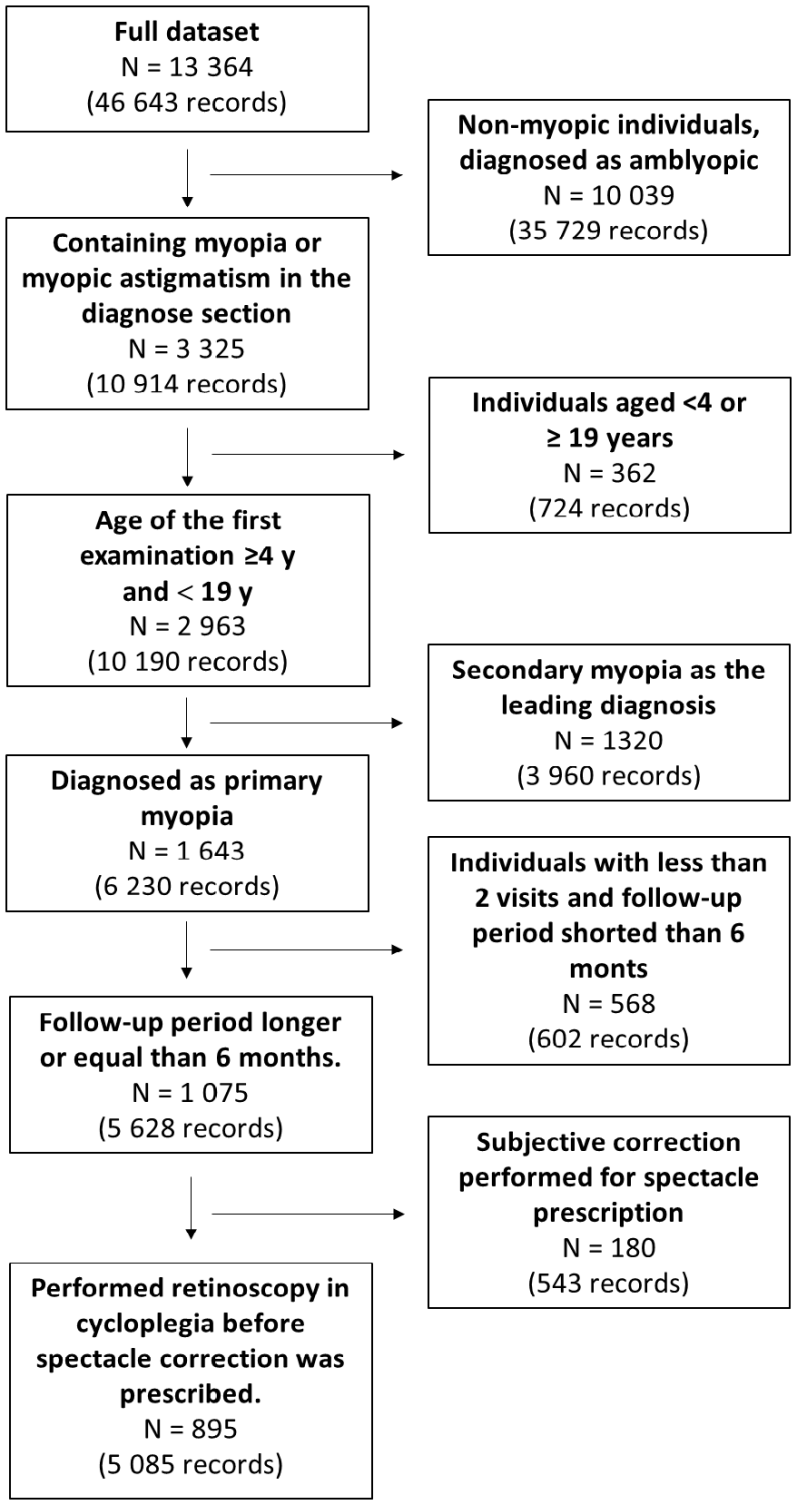
Flow diagram describing data selection.

The exclusion criteria were as follows: patients with eye comorbidities, including mixed astigmatism, strabismus, corneal diseases, retinopathy of prematurity, amblyopia, patients ≥19 years of age, and patients allergic to cycloplegic drugs. Detailed processing and exclusion steps are detailed in Figure 1.

Ethical approval was obtained from the ethics committee of the University Hospital “Sveti Duh”, Zagreb, and the School of Medicine of the University of Zagreb. The study adhered to the tenets of the Declaration of Helsinki.

### 2.2 Ophthalmic examination

Patients underwent a Standard Logarithmic Visual Acuity logMar inline chart test at near (40 cm) and at a distance (3 m) monocularly and binocularly without correction and with correction in diopters of spheres and cylinders and corresponding cylinder axis. Uncorrected visual acuity (UCVA) and best corrected visual acuity (BCVA) were measured with subjective refraction and visual acuity determined by retinoscopy in cycloplegia. Tropicamide 1% (Mydriacyl^®^, Alcon Laboratories Inc., Geneva, Switzerland) was instilled into each eye of the patient three times at intervals of 15 min to achieve the best possible mydriasis and cycloplegia.

A slit lamp examination and indirect examination of the fundi were performed to exclude eye comorbidities and secondary causes of myopia. Data from the legal guardians, as well as self-reported information about previous medical history, were also recorded for analysis, including history of previous ophthalmic diseases, surgeries, and parental myopia.

### 2.3 Definition of refractive status

Spherical equivalent (SE) was calculated as spherical power plus half of the cylindrical power. In accordance with the IMI definition, premyopia was defined as a condition in which the spherical equivalent refractive error of an eye is > −0.50 D and ≤ 0.75 D when ocular accommodation is relaxed. Myopia is classified as a condition in which the spherical equivalent refractive error of an eye is ≤ −0.50 and > −6.00 D when ocular accommodation is relaxed, whereas high myopia is a condition in which the spherical equivalent refractive error of an eye is ≤ −6.00 D when ocular accommodation is relaxed (7, 13). The SE of the worse eye was used to classify individuals into premyopia, myopia, and high myopia.

### 2.4 Statistical analysis and data modeling

Data analysis was performed using Graphpad Prism software version 8.0 (Graphpad software Inc., San Diego, CA, United States) and R software (version 4.0.3).^1^ Independent samples t-test and Kruskal–Wallis tests with the *post-hoc* test were applied to compare differences between groups. A multivariate Cox proportional hazards model was employed for survival analysis. The model incorporated several covariates, including age at baseline, baseline SE, parental history of myopia, and gender. These covariates were adjusted in relation to the duration of the follow-up period. Logistic regression analysis was conducted to predict individuals who, between 11 and 24 months after the first visit, would exhibit a rate of myopic progression that is equal to or exceeds −0.5 D/y. Myopia progression was defined as the difference in SE between subsequent SE and baseline SE. Negative values represent myopia progression. Time intervals between visits in the first 2 years were categorized into 6-month intervals, and after the 2nd year, the intervals were divided into yearly follow-ups. In case of multiple visits within the interval, the last visit was selected. Progression rates were calculated depending on the groups that were compared: average 1st-year progression rates for different years of first visit and myopia progression heatmap, average 3-year progression rates for different ages of first visit, progression rates between 11 and 24 months after the first visit for groups that were separated by age, and spherical equivalent at baseline, gender, and parental myopia.

## 3 Results

### 3.1 Dataset description

The dataset included 895 children and adolescents (51 premyopes, 813 low myopes, and 31 high myopes). On average, the sample included more girls (60.8% when the diagnosis was premyopia and 58.7% if low myopia was diagnosed). More boys (51.6%) were diagnosed as high myopes. The average age of diagnosis was 11.37 ± 3.59 years for premyopic individuals, 11.18 ± 3.53 years for low myopic, and 11.44 ± 4.35 years for high myopic individuals. Demographic characteristics and refractive data are detailed in Table 1.

**Table 1:** Sample demographic and refractive characteristics.

	*N*	Gender (female, %)	Age of diagnosis (mean ± SD, y)	Follow-up period (mean ± SD, y)	1st visit Cycloplegic SE (mean ± SD, D)	Last visit Cycloplegic SE (mean ± SD, D)
Premyopia	51	60.8	11.37 ± 3.59	2.81 ± 2.64	−0.22 ± 0.14	−1.06 ± 1.01
Low myopia	813	58.7	11.18 ± 3.53	2.97 ± 2.51	−1.82 ± 1.08	−2.56 ± 1.68
High myopia	31	48.4	11.44 ± 4.35	3.35 ± 3.14	−8.08 ± 1.92	−8.97 ± 2.15

The highest percentage in the follow-up visit was observed in low myopic individuals, with 602 (74%) participants followed up for ≥2 years and 90 (11%) participants for ≥7 years, as shown in Table 2.

**Table 2:** Number and percentage of follow-up visits in premyopic, low myopic, and high myopic individuals.

	Follow-up duration	≥2 years	≥3 years	≥4 years	≥5 years	≥6 years	≥7 years
Premyopia	Number of individuals	26	21	19	13	12	6
	Percentage	51	41	37	25	24	12
Low myopia	Number of individuals	602	521	399	317	178	90
	Percentage	74	64	49	39	22	11
High myopia	Number of individuals	27	20	16	9	6	5
	Percentage	85	62	52	29	19	16

### 3.2 Progression of premyopia, low myopia, and high myopia depending on the year and age of first visit

**Figure 2:**
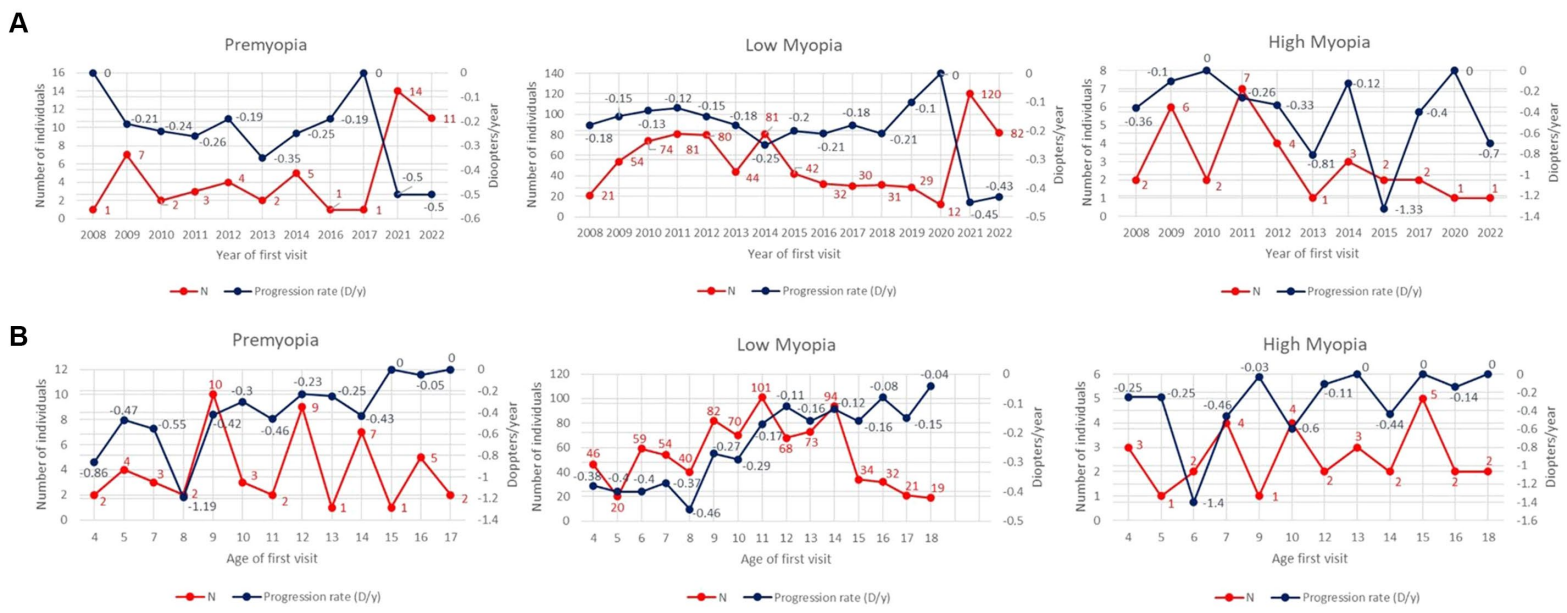
**(A)** Average 1st year progression rates (D/y) and total number of individuals as a function of the year of first visit through the period from 2008 till 2022. The left y-axis is related to the number of individuals (red line), and the right y-axis is related to progression rates (blue line). **(B)** Average 3-year progression rates (D/y) and total number of individuals as a function of the age of the first visit. The left y-axis is related to the number of individuals (red line), and the right y-axis is related to progression rates (blue line).

Comparing the total number and average 1st-year progression rates of individuals based on the year of first visit (2008–2022), an increase in the number of premyopic and low myopic individuals was noted after 2020, as shown in Table 3 and Figure 2A. Progression rates and number of individuals in 2020 were separated as a single year in Table 3 due to specific circumstances related to COVID-19 restrictions. In 2021, the highest number of premyopic (13) and low myopic (120) patients were detected. Average 1st-year progression significantly increased (*p* < 0.001) in both premyopic and low myopic individuals, with the most rapid progression being observed in 2021 and 2022, −0.5 ± 0.12 D/y for premyopes, and − 0.45 ± 0.1 D/y for low myopic individuals, as shown in Table 3. The progression rates of high myopic individuals exhibited a non-linear pattern, while the overall number of individuals showed a declining trend; however, both trends were not statistically significant (*p* = 0.862 and *p* = 0.562), as shown in Figure 2A and Table 3.

**Table 3:** Average 1st year progression rates (mean ± SD, D/y) as a function of the year of the first visit.

	Year of first visit	2008–2010	2011–2013	2014–2016	2017–2019	2020	2021–2022	*p*-value
Premyopia	*N*	10	9	6	1	0	25	**<0.001**
	Progression rate (mean ± SD, D/y)	−0.20 ± 0.11	−0.25 ± 0.08	−0.24 ± 0.08	0.00 ± 0.00	-	−0.50 ± 0.12	**<0.001**
Low myopia	N	149	205	155	90	12	202	**<0.001**
	Progression rate (mean ± SD, D/y)	−0.14 ± 0.08	−0.14 ± 0.05	−0.23 ± 0.08	−0.16 ± 0.04	0.00 ± 0.00	−0.45 ± 0.1	**<0.001**
High myopia	N	10	12	5	2	1	1	0.56
	Progression rate (mean ± SD, D/y)	−0.13 ± 0.15	−0.33 ± 0.24	−0.60 ± 0.72	−0.40 ± 0.10	0.00 ± 0.00	−0.70 ± 0.00	0.86

The Kruskal–Wallis tests with the *post-hoc* test were used to test the significance of differences between groups. Bolded values represent statistically significant results.

Older individuals diagnosed as premyopic and low myopic demonstrated slower (*p* < 0.001) progression rates than individuals diagnosed as premyopic and low myopic at a younger age. The slowest progression rates were observed in the age group from 16 to 18 years (−0.04 ± 0.05 D/y) in premyopic individuals and low myopic individuals at the age of 16–18 (−0.09 ± 0.06 D/y) years, as shown in Table 4. The highest progression rates were observed at the age of 8 years in both premyopic and low myopic individuals, as illustrated in Figure 2B. Individuals diagnosed as high myopic showed a decrease in progression rates with the age of diagnosis, as shown in Figure 2B and Table 4.

**Table 4:** Average 3-year progression rates (mean ± SD, D/y) as a function of the age of the first visit.

	Age of first visit	4–6 years	7–9 years	10–12 years	13–15 years	16–18 years	*p*-value
Premyopia	N	6	15	14	9	7	0.13
	Progression rate (mean ± SD, D/y)	−0.60 ± 0.31	−0.55 ± 0.41	−0.28 ± 0.13	−0.36 ± 0.21	−0.04 ± 0.05	**<0.001**
Low myopia	N	125	176	239	201	72	**<0.001**
	Progression rate (mean ± SD, D/y)	−0.38 ± 0.21	−0.34 ± 0.19	−0.19 ± 0.12	−0.14 ± 0.11	−0.09 ± 0.06	**<0.001**
High myopia	N	6	5	6	10	4	0.86
	Progression rate (mean ± SD, D/y)	−0.63 ± 0.66	−0.37 ± 0.30	−0.44 ± 0.36	−0.09 ± 0.26	−0.07 ± 0.09	0.96

The Kruskal–Wallis tests with the *post-hoc* tests were used to test the significance of differences between groups. Bolded values represent statistically significant results.

**Figure 3:**
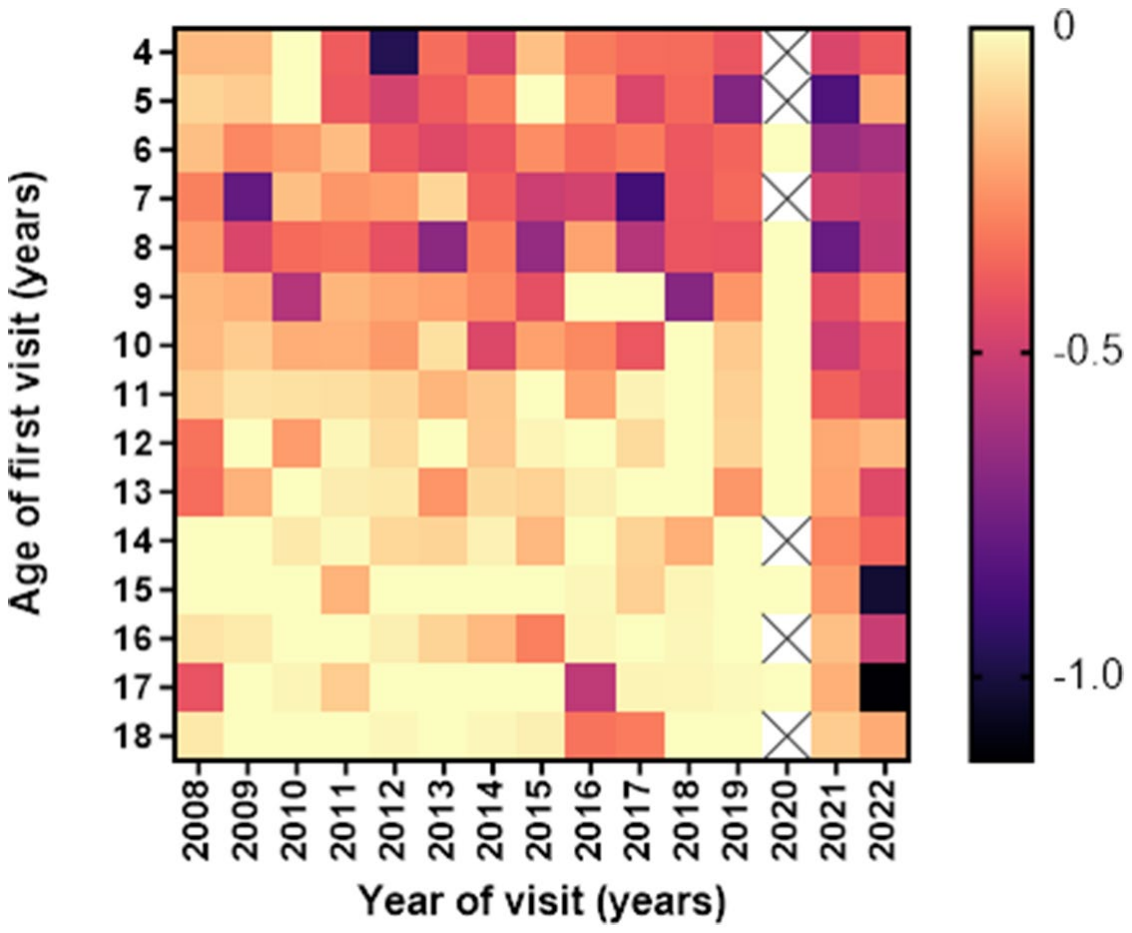
Heatmap of average 1st year low myopia progression (in diopters) according to age and year of the first visit. Darker colors indicate more rapid progression rates (D/y).

Children with low myopia had faster progression rates when diagnosed earlier than 10 years of age. The fastest progression rates in low myopic individuals in all age groups were observed in the years 2021 and 2022, as illustrated in Figure 3.

### 3.3 Progression of premyopia to low myopia as a function of progressor status

Premyopic individuals who progressed to low myopia were diagnosed younger (*p* < 0.001, CI −1.91 to −0.67), had one (33%) or both (43%) parents that were myopic (*p* < 0.001, 95% CI −2.42 to −1.09), and had a significantly longer follow-up (*p* = 0.02, 95% CI 0.08 to 1.22), while on the first examination, cycloplegic SE was similar in both groups (−0.22 ± 0.15 D), as shown in Table 5.

**Table 5:** Demographic and refractive characteristics of premyopic individuals based on the progression status to low myopia.

	Number of individuals	Gender (female, %)	Parental myopia (both/single, %)	Age of diagnosis (mean ± SD, y)	Follow-up period (mean ± SD, y)	1st visit Cycloplegic SE (mean ± SD, D)	Last visit Cycloplegic SE (mean ± SD, D)
Progressors	30	63%	43%/33%	9.75 ± 3.01	3.46 ± 3.01	−0.22 ± 0.15	−1.57 ± 1.03
Non-progressors	21	57%	9%/14%	13.68 ± 2.97	1.87 ± 1.65	−0.22 ± 0.15	−0.32 ± 0.08
*p*-value, 95% CI		0.67, −1.76 to −1.02	**<0.001, −2.42 to − 1.09**	**<0.001, −1.91 to − 0.67**	**0.02, 0.08 to 1.22**	0.98, −0.56 to 0.55	**<0.001, −2.44 to − 1.02**

An independent sample *t*-test was used to test the significance of differences between the two groups. Bolded values represent statistically significant results.

The multivariate analysis demonstrated that the highest hazard ratio of developing low myopia is in individuals with both and a single myopic parent (920.27 and 437.38), while younger children and adolescents showed an increased risk of developing low myopia, as shown in Table 6.

**Table 6:** Hazard ratios and 95% CI for the event “develops low myopia” from a multivariate Cox model.

	*N*	Multivariate HR (95% CI)^a^
**Age**		*p*-value = 0.02
4–6	6	1.38 (0.85 to 1.88)
7–9	15	2.42 (1.53 to 3.21)
10–12	14	1.21 (0.55 to 1.72)
13–15	9	1.02 (0.75 to 1.22)
16–18	7	Reference
**Gender**		*p*-value = 0.26
M	20	Reference
F	31	1.21 (0.48 to 3.02)
**Parental myopia**		*p*-value =0.01
Both parents	15	920.27 (850.16 to 950.53)
One parent	13	437.23 (396.57 to 453.79)
Non-myopic parent	23	Reference

Type 3 test *p*-values are displayed.

a Adjusted to the duration of follow-up time as a covariate.

### 3.4 Progression of low myopia as a function of age and SE on the first visit, parental myopia, and according to gender

**Figure 4:**
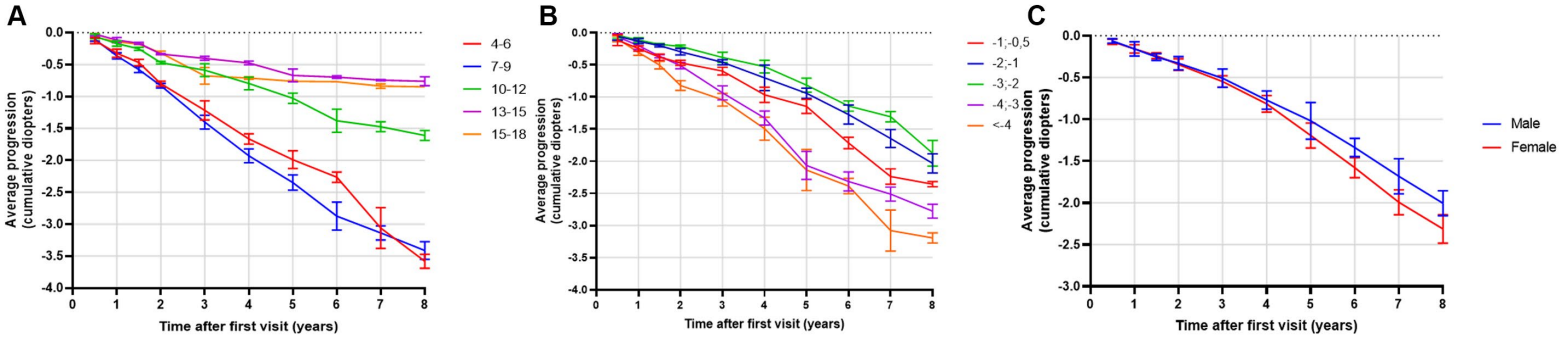
**(A)** Average progression of low myopia (in diopters) as a function of time (in years) stratified by baseline age groups. Bars display 95% CIs. **(B)** Average progression of low myopia (in diopters) as a function of time (in years) stratified by baseline spherical equivalent (SE). Bars display 95% CIs. **(C)** Average progression of myopia (in diopters) as a function of time (in years) stratified by gender. Bars display 95% CIs.

**Figure 5:**
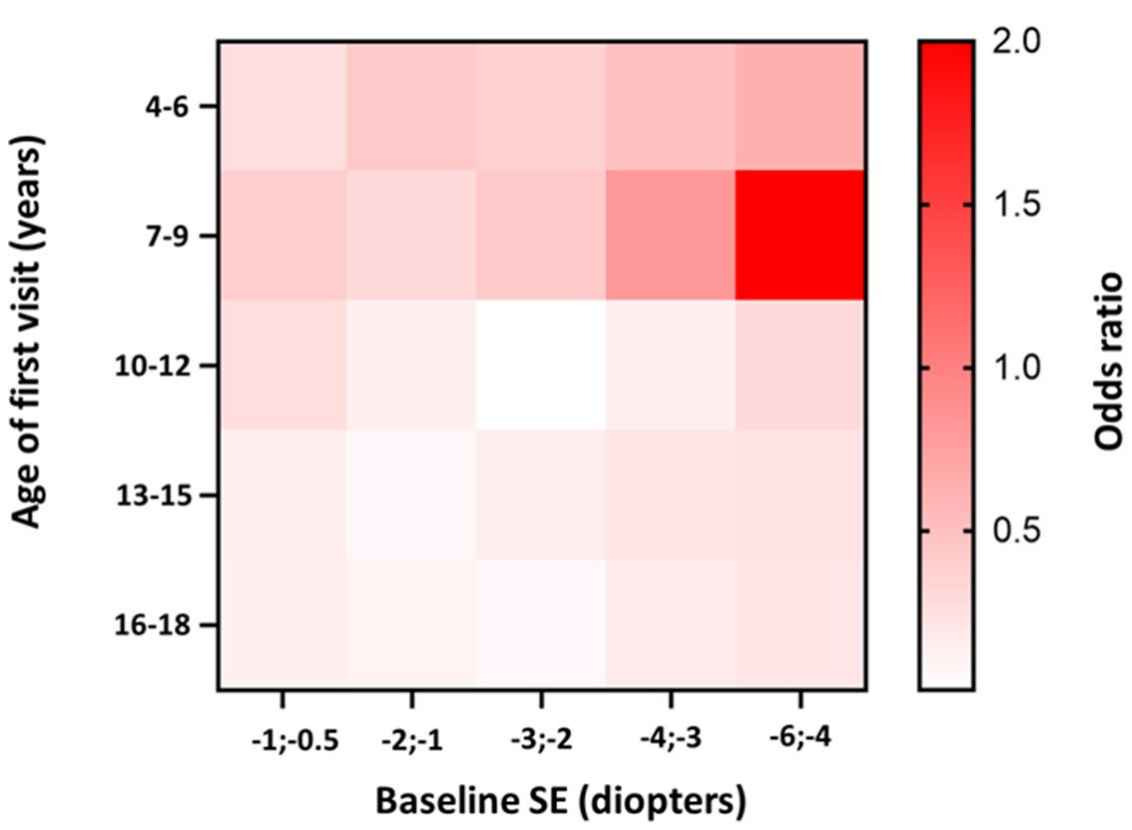
Heatmap of low myopia individuals odds ratio to progress faster or equal −0.5 D/y in the period between 11 and 24 months after the first visit, stratified by age and baseline SE. Darker tones indicate higher OR values. The *p*-value for the model is <0.001.

The highest progression rates in the period between 11 and 24 months after the first visit were observed in individuals with both myopic parents, −0.69 D/y, as shown in Table 7. Regarding age groups, low myopic individuals aged between 7 and 9 years and between 4 and 6 years displayed the fastest myopia progression rates, as shown in Table 7 and Figure 4A. Individuals older than 13 years experienced the slowest progression rates, −0.10 D/y, as shown in Table 7 and Figure 4A, with almost no progression after 5 years of follow-up, as illustrated in Figure 4A. Children with SE > −1 D demonstrated faster progression rates than children with SE between −1 D and − 3 D, as shown in Table 7 and Figure 4B. Among girls, the progression rates of low myopia were higher, −0.21 D/y, compared to boys, −0.18 D/y, as indicated in Table 7. Additionally, Figure 4C illustrates that the difference between girls and boys in progression was more prominent with a longer follow-up. By segmenting low myopes based on the progression rates of myopia from 11 to 24 months after the first visit, individuals diagnosed with low myopia at ages 7–9 years, and with baseline SE between −6 D and − 4 D were strongly associated with progression of ≤ − 0.5 D, with an OR = 2.0 (95% CI −1.00 to 2.39), as illustrated in Figure 5.

**Table 7:** Average low myopia progression rates (in diopters) between 11 and 24 months according to age, spherical equivalent at baseline, gender, and parental myopia using multivariate analysis.

		Progression rate		
	*N*	Mean (D/y)	95% CI MU	95% CI ML
**Age**				
4–6	109	−0.29	−0.18	−0.39
7–9	154	−0.36	−0.24	−0.45
10–12	207	−0.17	−0.11	−0.22
13–15	175	−0.10	−0.04	−0.15
16–18	105	−0.10	−0.03	−0.22
**SE**				
−1; −0.5	98	−0.23	−0.12	−0.34
−2; −1	305	−0.19	−0.13	−0.24
−3; −2	182	−0.10	−0.04	−0.13
−4; −3	71	−0.29	−0.15	−0.14
≤ −4	94	−0.34	−0.13	−0.12
**Gender**				
F	449	−0.21	−0.14	−0.27
M	301	−0.18	−0.13	−0.23
**Parental myopia**				
Both parents	46	−0.69	−0.52	−0.87
One parent	263	−0.31	−0.27	−0.35
Non-myopic parent	441	−0.13	−0.11	−0.15

Type 3 test *p*-values for the multivariate model: age (*p* < 0.001), spherical equivalent (SE) (*p* < 0.001), gender (*p* = 0.04), and parental myopia (*p* < 0.001).

MU, confidence interval upper bound; ML, confidence interval lower bound.

### 3.5 Progression of high myopia as a function of different progression rates

Individuals diagnosed with high myopia were divided into two distinct groups depending on the speed of progression: the fast progressors with progression rates ≤ −0.5 D and slow progressors > −0.5 D/y. A significant difference between the two groups was observed in the age of diagnosis (0.04, −0.04 to 1.77); fast progressors were diagnosed at a younger age (8.80 ± 3.28 years) compared to slow progressors (12.20 ± 4.38 years), as shown in Table 8.

**Table 8:** Demographic and refractive characteristics of high myopic individuals based on different progression rates.

	Number of individuals	Gender (female, %)	Parental myopia (both/single, %)	Age of diagnosis (mean ± SD, y)	Follow-up period (mean ± SD, y)	1st visit Cycloplegic SE (mean ± SD, D)	Last visit Cycloplegic SE (mean ± SD, D)
Slow progressors (> −0.5 D/y)	24	45.8	8%/41%	12.20 ± 4.38	3.47 ± 3.32	−7.97 ± 1.85	−8.35 ± 1.83
Fast progressors (≤ −0.5 D/y)	7	57.1	42%/28%	8.80 ± 3.28	2.94 ± 2.32	−8.43 ± 2.26	−11.09 ± 1.90
*p*-value, 95% CI		0.63, −1.06 to 0.64	0.16, −1.60 to −0.22	**0.04, −0.04 to 1.77**	0.64, −0.66 to 1.03	0.64, −0.64 to 1.06	**0.01, 0.38 to 2.51**

Independent sample *t*-test was used to test the significance of the difference between the two groups. Bolded values represent statistically significant results.

### 3.6 Progression of low myopia to high myopia

**Figure 6:**
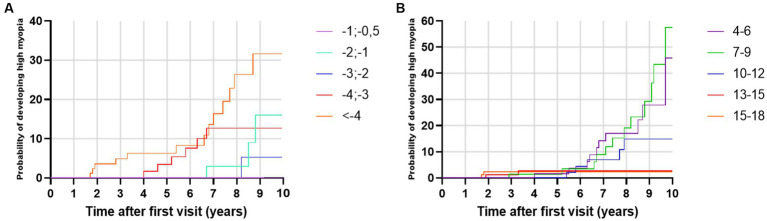
**(A)** Kaplan–Meier failure curves for the event ‘develops high myopia’ stratified by spherical equivalent at baseline. **(B)** Kaplan–Meier failure curves for the event ‘develops high myopia’ stratified by baseline age groups.

A total of 32 low myopes developed high myopia in the period of 10 years after the first visit. Univariate analysis showed that low myopic individuals with SE ≤ −4 D at baseline had a 32.78% risk of developing high myopia in the period of 10 years of diagnosis, while individuals with SE ≤ −0.5 D and > −1.0 D had a 0% risk of developing high myopia in the observed period, as shown in Figure 6A. Multivariate analysis demonstrated that young girls with lower SE and myopic parents were more likely to develop high myopia, as shown in Table 9.

**Table 9:** Hazard ratios and 95% CI for the event “develops high myopia” from a multivariate Cox model.

	*N*	Multivariate HR (95% CI)^a^
**Age**		*p*-value <0.001
4–6	125	2.04 (1.76 to 2.21)
7–9	176	2.53 (1.34 to 3.21)
10–12	239	1.11 (0.85 to 1.41)
13–15	201	Reference
16–18	72	1.03 (0.82 to 1.29)
**Spherical equivalent**		*p*-value <0.001
(−1; −0.5)	100	Reference
(−2; −1)	330	8.73 (6.14 to 11.25)
(−3; −2)	209	6.37 (4.28 to 8.72)
(−4; −3)	73	64.28 (30.89 to 99.23)
(−6; −4)	101	287.56 (210.85 to 376.78)
**Gender**		*p*-value <0.001
M	335	Reference
F	478	1.05 (1.01 to 1.10)
**Parental myopia**		*p*-value <0.001
Both parents	51	199.23 (141.10 to 250.65)
One parent	285	123.45 (91.45 to 156.78)
Non-myopic parent	477	Reference

Type 3 test *p*-values are displayed.

a Adjusted to the duration of follow-up time as a covariate.

## 4 Discussion

To the best of our knowledge, this is the first organized database on premyopia, low myopia, and high myopia in the Republic of Croatia. Analyzing data from individuals aged 4–18 years at baseline and followed in the period from 2008 to 2023 at the University Eye Department of the University Hospital “Sveti Duh,” Zagreb, the CroMyop™ registry for the first time described the proportion patterns and progression rates of premyopia, low myopia, and high myopia throughout years of follow-up and different ages of myopia diagnosis.

High prevalence and incidence of myopia in Asian children and adolescents have been reported in the past few decades (16), resulting in a large number of data regarding myopia progression (17–19). Recently, there has been an increased number of published data on myopia progression in European children (14).

In accordance with the Studies on Health of Children and Adolescents (KiGGS studies) (20), our data confirmed that the total number of myopic children and adolescents did not meaningfully change between 2008 and 2019. However, an increased number of myopic patients during and after the COVID-19 pandemic was reported. Several myopia studies have demonstrated an increase in the incidence of myopia in connection with the COVID-19 pandemic (21–31). Data on European children demonstrated that in England, after the pandemic, a reduction of visual acuity in children was recorded, suggesting an increase in myopia progression (25). Slovakian, Russian, and Spanish authors demonstrated a decrease in spherical equivalent and an increase in the myopia prevalence and axial length of eyes in children following the COVID-19 pandemic compared to the period preceding the pandemic (25–31). During the COVID-19 pandemic, habitual changes affecting myopia progression in children were studied by Xu et al. (24). The progression of myopia over a period of 6 months worsened from −0.23 D to −0.34 D, and the incidence of myopia doubled, from 8.5 to 13.6% (24). In our study, myopia progression rates in the years 2017 and 2019 varied from −0.1 D/y to −0.21 D/y; however, in 2021 and 2022, an increase in myopia progression was observed, −0.45 D/y and − 0.43 D/y. The number of premyopic and low myopic children and adolescents increased significantly. Heatmaps of average first-year progression rates indicate faster progression rates in all age groups during COVID-19 and post-COVID-19 years. This increase in myopia prevalence and progression could be explained by the reduction in outdoor activities combined with an increase in close-range work activities, digital screen time, and homeschooling, which are proven risk factors for myopia onset and progression (32).

Together with environmental factors, described risk factors for faster myopia progression are myopic parents (33), higher myopia at baseline, age between 7 and 12 years, and female gender (14, 34). Parental myopia significantly increases the risk of high myopia (33), myopia onset, and progression (35, 36). Myopia prevalence was significantly linked with paternal history of myopia (OR = 2.4). The Finnish study noted that both parents were myopic in 54% of cases, and the OR of developing high myopia was 3.96 compared to non-myopic parents (33). In Bulgaria, 23.46% of myopic children had at least one myopic parent, leading to a significant risk factor for myopia onset (37). In Croatia, parents of myopic children and adolescents were mostly non-myopic, but a high degree of individuals had one myopic parent (35%). If both parents were myopic, hazard ratios for developing low myopia from premyopia and high myopia from low myopia were 920.27 and 199.23 compared to individuals without myopic parents. Progression rates between 11 and 24 months after the first visit were fastest among children and adolescents with both myopic parents (−0.69 D/y). However, such observations should not be associated with just genetic inheritance but also with the family lifestyle and habits, such as outdoor activity, use of digital screens, time spent near work, daily sports training, and time spent with children in general (37). Genetic variations cannot fully explain the majority of variations in myopia risk, hence the known genetic loci from genetic exploration in the recent 20 years only explained less than 20% of the heritability for myopia in European children (38, 39).

Our study demonstrated that the mean age of diagnosis for premyopia, low myopia, and high myopia was between 11 and 12 years of age, which is similar to findings from French and Irish studies (14, 36). Regarding progression rates, age was identified as the most important risk factor for faster myopia progression (10, 40, 41). Children from 7–9 years demonstrated the highest progression rates (−0.36 D/y). Heatmaps of low myopia individuals’ odds ratio to progress faster or equal −0.5 D/y in the period between 11 and 24 months showed that the 7–9 age group with baseline SE from −4 D to −6 D demonstrated the strongest association with faster progression (OR = 2.0, 95% CI −1.00 to 2.39). Furthermore, younger individuals had a higher risk of developing low myopia from premyopia and high myopia from low myopia. High myopia progression rates illustrated statistically significant differences between younger and older individuals, where 8-year-old children progressed more than 0.5 D/y compared to 12-year-old individuals with high myopia. School starting age in Croatia is around 7 years, which is in accordance with the fastest progression rate of premyopia and low myopia at the age of 8 years (−1.19 D/y and − 0.46 D/y). Our data are comparable to other studies that included children from 4 to 18 years of age. The French study revealed that the same age group from 7 to 9 years progressed the fastest (−0.43 D) (14), while progression reported in the control arm of children aged 6–15 years of the Houston Myopia Control Study in the youngest age group was −0.34 D/year (42). Differences were noted in other age groups, especially low myopic individuals aged 4–6 years, who progressed second fastest (average progression rate – 0.29 D/y). Progression in myopic individuals in France for the 4–6 years age group was −0.18 D (14). Myopic children and adolescents in the UK, with an average age of 5.6 years, progressed at a rate of −0.30 D/y (43). Different progression rates can be explained by differences in lifestyle and educational practices and socioeconomic factors, including access to healthcare, awareness of eye health, public health initiatives, availability of corrective measures such as spectacles or contact lenses, cultural attitudes toward outdoor activities, near-work habits, and the emphasis on academic achievements can vary from one country to another resulting in different myopia progression rates. Furthermore, the age groups from 13 to 18 years showed the slowest progression rates. This phenomenon might be attributed to the observation that the average age of myopia stabilization typically falls within this age bracket (11).

An important factor in myopia progression rate is ethnicity, demonstrating faster progression rates (−0.63 D/y to −2.00 D/y) in children of East Asia (12). Our population of children and individuals was ethnically homogeneous, which helps in a better understanding of myopia progression in Caucasian European children.

The ratio of individuals progressing from low to high myopia was around 3%, emphasizing the risk assessment of developing high myopia in different baseline SE groups. Lower SE groups at baseline proved to have higher hazard ratios than higher baseline SE groups. Our finding was in concordance with observations from other European studies (14, 33), suggesting refractive characteristics of individuals that should be more often followed up due to a higher risk of developing high myopia (14).

Although girls’ progression rates are faster than in boys (−0.21 D/y vs. −0.18 D/y), it is not clinically meaningful. In China, United Kingdom, and France, progression rates in girls were faster than in boys, but these observations were equally clinically unmeaningful (14, 41, 44) and usually a common finding (44).

This is the first Croatian study on myopia that included a large sample of individuals and respective records of visits. The strength of this study is the clinical setup where all examiners were experienced pediatric ophthalmologists performing a complete ophthalmological examination, including retinoscopy in cycloplegia, excluding individuals with secondary myopia. Children aged 4–18 years, in the period from 2008 to 2023, were diagnosed with myopia and followed up. The study included Caucasian children and adolescents from Croatia and surrounding countries, featuring an examination of both eyes, with the worst eye refraction status analysis. Individuals were prescribed spectacle correction without any other interventions affecting the sight. Regarding the size of the population and number of children in the Republic of Croatia, we analyzed data from 3% of children and adolescents, filtering data to approximately 0.3% of children and adolescents with primary myopia who underwent cycloplegia on the first examination and followed up longer than 6 months. In comparison, the number of children and adolescents included in the study regarding the population of children and adolescents in a country was France 2.3% (14), United Kingdom 0.3% (43), Germany 0.5% (20), Spain 0.1% (45), United States 0.01% (44), India 0.1% (46), and China 0.2% (23). By performing a complete ophthalmic examination and retinoscopy in cycloplegia, we managed to exclude secondary myopia and other causes that can lead to misinterpretation of the results. We also acknowledge several limitations in this study. Children with constant myopia progression could be over-represented, given that they would require more frequent changes of optical correction than those with more stable vision. This may explain a paradox in the data: while it is clear that older children progress more slowly, older age groups do not appear to slow over time. Retrospective data collection limited the analysis of biometrical eye data, which would help in better understanding of progression rates in some groups. A standardized classification of myopia defining premyopia was proposed in 2018 (9), and excluded children with cycloplegic SE ≥ 0 D examined in the years before 2018. Progression rates of children diagnosed with myopia, in 2021 and 2022 and followed till June 2023, could be affected by a shorter follow-up period. Furthermore, the retrospective design of the study resulted in a loss of follow-up over the years. Additionally, analyzing cycloplegic SE in children who were examined and followed up for spectacle prescription may have influenced progression rates, while small refractive changes that did not express a need for new spectacle prescription, did not include retinoscopy in cycloplegia, or possibly were not reported at the end leading to steep changes in cycloplegic SE.

## 5 Conclusion

This is the first Croatian database analyzing premyopic, low myopic, and high myopic Caucasian children and adolescents aged 4–18 years and followed in the period from 2008 to 2023, providing data on myopia progression according to age, gender, parental myopia, and initial degree of myopia at first correction, based on cycloplegic refraction.

## Data availability statement

Anonymized data is available upon reasonable request. Please direct any requests to access the dataset to the corresponding author.

## Ethics statement

The studies involving humans were approved by Ethics committee of University Hospital “Sveti Duh”, Zagreb, Ethics committee of the School of Medicine of the University of Zagreb. The studies were conducted in accordance with the local legislation and institutional requirements. Written informed consent for participation was not required from the participants or the participants’ legal guardians/next of kin in accordance with the national legislation and institutional requirements.

## Author contributions

AV: Conceptualization, Data curation, Formal analysis, Investigation, Methodology, Project administration, Validation, Visualization, Writing – original draft. LM: Conceptualization, Data curation, Formal analysis, Investigation, Software, Validation, Writing – review & editing. ZS: Funding acquisition, Project administration, Resources, Software, Supervision, Writing – review & editing.

## Footnotes

^1^ https://www.r-project.org/
